# Supplementary material for: Drinking Water Supplemented with Acidifiers Improves the Growth Performance of Weaned Pigs and Potentially Regulates Antioxidant Capacity, Immunity, and Gastrointestinal Microbiota Diversity
Source: Antioxidants (Basel). 2022 Apr 21;11(5):809. doi: 10.3390/antiox11050809 (PMC9138078; doi:10.3390/antiox11050809)
Supplement: Supplementary file 1 [file antioxidants-11-00809-s001.zip › Supplemental Tables.pdf]

Tables

Table S1 Experimental diet composition and nutrients (as-fed basis, %)

Item	Content
Ingredients	
…Corn	54.0
…Soybean meal	23.0
…Whey power	10.00
…Fish meal	6.00
…Glucose	2.00
…Yeast	2.00
…Limestone	0.9
…Soybean oil	0.70
…$CaHPO_4$	0.4
…Nacl	0.3
…L-lys	0.1
…Zinc oxide	0.1
…Vitamin premix[1]	0.5
Calculated nutrients	
…Digest energy, Mcal/kg	3.50
…Crude protein	20.0
…Digestible lysine	1.50
…Crude Ash	7.0
…Available phosphorus	0.55
…Available Ca	0.5-1.2
Nacl	0.3-0.8

[1]Vitamin premix provided the following per kilogram diet: vitamin A, 8.5 MIU; vitamin D, 1,900 IU as vitamin D3; vitamin E, 24 IU, vitamin K3, 2.2 mg, vitamin B1 2 mg, vitamin B2 5 mg, vitamin B6 3 mg, vitamin B12 0.03 mg, vitamin B3 24 mg, vitamin B5 12 mg, vitamin B9 1 mg, vitamin B7 0.1 mg, vitamin C 36 mg.
